# Parents’ knowledge, beliefs, and acceptance of the HPV vaccination in relation to their socio-demographics and religious beliefs: A cross-sectional study in Thailand

**DOI:** 10.1371/journal.pone.0193054

**Published:** 2018-02-15

**Authors:** Maria Grandahl, Seung Chun Paek, Siriwan Grisurapong, Penchan Sherer, Tanja Tydén, Pranee Lundberg

**Affiliations:** 1 Department of Women’s and Children’s Health, Uppsala University, Uppsala, Sweden; 2 Department of Society and Health, Mahidol University, Salaya Campus, Bangkok, Thailand; 3 Department of Public Health and Caring Sciences, Uppsala University, Uppsala, Sweden; Rudjer Boskovic Institute, CROATIA

## Abstract

Thailand has one of the world’s highest prevalence of cervical cancer, mainly caused by the human papillomavirus (HPV). HPV infections can successfully be prevented by vaccination, which is available at a cost but not yet implemented in the national vaccination program. Parents play a critical role in deciding whether to vaccinate their child against HPV. Thus, the aim was to examine the association between parents’ knowledge, beliefs, and acceptance of the HPV vaccination for their daughters, considering their socio-demographics and religious beliefs. A cross-sectional design was used among three schools in Thailand: Nakorn Phatom province (suburban) and Bangkok (urban). Parents of 9–12-year-old daughters completed the questionnaires, guided by the Health Belief Model. In total, 359 parents completed the questionnaires; of those, 301 were included in the final analyses. The ordinary least squares (OLS) regression analysis showed that background knowledge of HPV and the HPV vaccine was positively related to knowledge of HPV and cervical cancer. For beliefs, knowledge was positively associated with susceptibility (i.e., parents’ perceived risk of an HPV infection/ related disease), severity, and benefit. However, knowledge was not significantly related to barriers. For acceptance, higher susceptibility and benefit were related to higher acceptance, and greater knowledge was associated with higher acceptance. Thus, we found associations between parents’ knowledge, beliefs, and acceptance of the HPV vaccination for their daughters, considering their socio-demographics and religious beliefs. Parents, who reported religion as important, as opposed to those who did not, were more favorable toward the HPV vaccination. Four out of ten mothers had never undergone a cervical cancer screening, but most had accepted previous childhood vaccinations for their daughters. The overall acceptance of the vaccine was high, and we believe our results are promising for future implementation of the HPV vaccination in the national childhood vaccination program in Thailand.

## Introduction

Human papillomavirus (HPV) infections are the world’s most common sexually transmitted infection (STI) among women and men; the estimated lifetime probability of getting an infection is over 80% [1]. HPV can cause condyloma, and some HPV types are associated with cancer in the oropharynx, vagina, anus, and penis. Globally, cervical cancer is the most common cancer caused by HPV; approximately 270,000 women die due to the disease annually, although cancer in the head and neck is rapidly increasing [2]. In Thailand, cervical cancer is the second most common cancer; approximately 8,200 women are diagnosed (incidence) every year, and more than half of these women, i.e., more than 4,500, die annually due to the disease, the majority under age 44 [3].

HPV-related diseases can successfully be prevented by primary prevention, the prophylactic vaccination. HPV can also be prevented by correct and consistent condom use. Secondary prevention includes early detection of abnormal cell changes with regular cervical cancer screening [4–7]. Worldwide, HPV vaccine is implemented in national vaccination programs, mainly for young girls. However, the vaccine is approved for both genders, and some countries and regions also include boys [8, 9].

Parents have an important role in the decision-making process regarding the HPV vaccination for their child. HPV vaccination was initially promoted as a vaccine protecting against a female disease, cervical cancer. This is reflected by the high proportion of female respondents, i.e., mothers, in studies about parents’ attitudes and beliefs about the HPV vaccination [10–14]. Moreover, studies indicate gender differences regarding the decision and knowledge about HPV and the HPV vaccination. Mothers are primary decision-makers for their daughters, and men have a lower level of knowledge about HPV and the HPV vaccination, especially about the link between HPV and oral and anal cancer compared to the link between HPV and cervical cancer [15–19].

Previous studies show that several factors are associated with the acceptance of the vaccine: knowledge, individual beliefs, and health behavior. Studies undertaken in different contexts show that parents’ knowledge about HPV and the HPV vaccination is low overall [20–25]. Positive parental beliefs and attitudes are important predictors of getting the HPV vaccination. Parents who perceive more benefits and believe that the HPV vaccination is efficient and that it protects against a severe disease are more likely to vaccinate their child [10, 11, 20, 26]. Moreover, previous acceptance of childhood vaccinations is associated with vaccine acceptance [10, 20, 27].

On the other hand, reasons as to why parents are reluctant to give consent for the vaccination include concerns about side effects and long-term safety of the vaccine, insufficient information prior to making informed consent, and the daughter’s young age [10–12, 27–29]. Other common barriers are low perceived risk of getting an HPV-infection or HPV-related cancer [10, 27, 28, 30]. There is a difference in vaccine initiation and completion, depending on one’s health behavior and socio-demographics (such as age, gender, religion, education level, employment/unemployment, income, and number of children). Moreover, lower socio-economics (education level and income) correlate with lower HPV vaccine uptake [31–33].

Cultural norms, moral values, and religious beliefs are other factors associated with the decision on whether or not to get the HPV vaccination [28, 34–36]. Religion, in particular, is a complex and multifactorial factor, and the specific religious perspective is likely to be different. Studies examining parents’ beliefs about the HPV vaccination in Brazil, Canada, Indonesia, the U.K, Sweden, and the U.S. show diverse and inconsistent results as to whether religion (Christian protestants, Catholic, Jewish, Hindu, and Muslim) has an impact on the decision to accept or decline the HPV vaccination [10, 20, 34, 35, 37–40]. An Internet based survey among parents in the U.S. found that Catholic parents were more likely to vaccinate their child than non-affiliated parents. However, religion has also been viewed as a barrier, and parents who attend religious services frequently were more likely than parents who did not attend services to decide against the HPV vaccination [34]. A cross-sectional study among Indonesian parents found that religion did not influence attitudes toward the HPV vaccination regardless of the parents’ religious affiliation, albeit Christian, Hindu, or Muslim [39]. Similar results have been found in studies undertaken in Sweden and Canada [10, 20]. This has also been supported by a cross-sectional study showing that religion did not correlate with compliance to vaccination among Catholic or protestant parents in Brazil [38]. In contrast, a qualitative study undertaken in the U.K. shows that religion was a barrier for the HPV vaccination among mothers in a Jewish community [35]. However, these studies are difficult to compare due to different methodological approaches and them being undertaken in different contexts with different aims.

In Thailand, vaccinations are provided free of charge as part of the national childhood vaccination program. Parents generally accept the recommended vaccinations, and the coverage is over 95%. Thailand is a Buddhist country, and religion has a substantial impact on all aspects of the society: the healthcare system and the regulations, cultural norms as well as individual health beliefs. The burden of HPV in Thailand is high, and the HPV vaccine is available at a cost but not included in the national vaccination program [41–43]. Parents must give consent to have their children be vaccinated, and their beliefs are of great importance for successful implementation. Thus, the aim of this study was to examine parents’ knowledge, beliefs, and acceptance of HPV and the HPV vaccine for their young daughter/s, in relation to their socio-demographics, health behavior, and religious beliefs in the Thai setting.

## Methods

### Theoretical framework

The theoretical framework used is the Health Belief Model (HBM). The HBM has previously been used in studies about parents’ beliefs about HPV and the HPV vaccination [20, 29, 44, 45]. According to the HBM, a person’s health behavior can be explained by the individual’s beliefs regarding the health action. The HBM includes these central constructs: *perceived benefit*, *perceived barriers*, *perceived susceptibility*, *and perceived severity* and also *cues to action*. Knowledge and socio-demographic variables such as age, sex, religion, and ethnicity as well as the education level and income are modifying factors for the individual behavior. Moreover, it is important for a person with risk behaviors to recognize the risk in order to be able to change his or her behavior. The benefits have to outweigh the barriers for a person to act upon the health promotion, for example, participating in a screening (cervical cancer) or vaccination program. The main limitation of the model is that it does not consider emotional aspects involved in an individual’s decision regarding their health behavior [46].

Our theoretical framework is based on previous research and clinical experiences. Socio-economic, cultural and environmental conditions, the community and social network, including work and health services, and individual lifestyle factors have an impact on health status. Health inequalities are related to socio-economic status and life conditions overall. Lower socio-economic (i.e., lower educational level and lower income) status is related to a poorer standard of health. Alcohol consumption, tobacco use, risky sexual behavior, and lack of adherence in attending recommended medical screening programs, such as cervical cancer screening, are associated with unhealthy behavior and risk for cancer. Addressing access to free and adequate healthcare services and changing health behaviors are important factors for reducing unhealthy behaviors as well as reducing the burden of cancer worldwide [47, 48].

Based on the HBM and literatures we reviewed, it was hypothesized that there would be differences among parents due to the demographic variables; parents with higher socio-economic status 1) had greater knowledge, 2) perceived more benefits, 3) perceived less barriers for the HPV vaccination, and 4) had higher HPV vaccine acceptance compared with parents with lower socio-economic status. It was also hypothesized that the importance of religion was associated with knowledge, beliefs, and acceptance of the HPV vaccination.

### Study design, sample, and setting

We used a cross-sectional approach; thus, this design cannot provide information about causality regarding the relation between parental attitudes, beliefs, behavior, knowledge, or acceptance of the HPV vaccination. Consequently, no experiments were conducted, and we cannot provide the causal relationship. Measurements of the parents’ attitudes, beliefs, knowledge, and acceptance of HPV and the HPV vaccination were undertaken on one occasion. Thus, when referring to relation throughout the paper, we do not point out the causality but an association between the variables.

The data were collected using a self-reporting questionnaire. A purposive sample of parents of daughters aged 9–12, with the ability to read and write in Thai, were invited to participate. Data were collected between September and November 2015 in three schools. Two schools in Bangkok as representative of the city (urban area) and one school in Nakhon Pathom Province as representative of the suburbs of Bangkok were selected. A combination of city and suburban schools was included to ensure the various enrolments of pupils living in low- and high- income areas. All classes from grades 3–6 in each school were recruited, targeting the parents of all female pupils. Inclusion criteria were being a self-identified parent or a primary caregiver of female pupil(s) aged 9–12-years-old in the schools and classes, as described above. The Health Belief Model [46] was used as the theoretical framework. The study follows reports of cross-sectional studies according to STROBE (S1 File.) [49].

### Procedure

One of the researchers (PS) contacted the principals at the respective schools and asked for permission to conduct the study. The principals received information about the study’s aim and procedure. After getting the ethical approval and permission from the schools, a research assistant who had been trained in collecting the data contacted the teachers, who were responsible for all the classes in grades 3–6 in each school, to schedule an appointment to give information about the study and deliver the questionnaires. The teachers delivered the informational letter about the study, the rights for participation, and the questionnaires to all female students in the class with the instruction to give to her mother or father (i.e., legal guardian) to complete. Parents of female students in the three schools were provided with written information, including that participation was voluntary and confidential. Those who agreed to participate completed the questionnaire at home. After three weeks, the research assistant contacted the teachers to collect the questionnaires. In total, 359 questionnaires were distributed: 186 in the two schools in Bangkok and 173 questionnaires in the school in the Nakhon Pathom Province.

### Questionnaire

The validated questionnaire (S2 File.) was based on previous studies [20, 50, 51] and comprised 18 principle questions (60 items) grouped into four areas: 1) Socio-demographic variables and health behavior (13 items); 2) knowledge related to HPV, the HPV vaccine, and cervical cancer (22 items); 3) beliefs about the HPV vaccine (16 items); and 4) acceptance of the HPV vaccination (9 items). In addition, one open-ended question for additional comments was included.

A five-point Likert scale was used for questions regarding beliefs about HPV and the HPV vaccine in relation to the HBM, with possible responses ranging from “strongly disagree” to “strongly agree.” A five-point scale was also used for questions about acceptance of the HPV vaccine. The questions regarding knowledge about HPV and the HPV vaccine had the response alternatives dichotomized: yes/no or true, false, or do not know. The remaining questions had multiple-choice alternatives. It took about 15–20 minutes to complete the questionnaire.

A pre-test was performed among 20 parents of female students aged 9–12-years-old before the actual data collection commenced. Some questions in the questionnaire were rewritten, i.e., clarified in order to make it clearer for the participants and increase the face- and construct validity.

### Variables and measurement

A total of six dependent variables were measured as continuous variables and used for the analysis, including (1) knowledge of HPV and cervical cancer, (2) susceptibility, (3) severity, (4) benefit, (5) barriers of HPV and the HPV vaccine, and (6) acceptance of the HPV vaccination. For knowledge of HPV and cervical cancer, each individual respondent was asked to answer a total of fourteen true or false questions, and the total number of correct answers was represented as the level of knowledge of HPV and cervical cancer. The value of the knowledge ranged from zero to fourteen, and a higher value indicated a higher level of knowledge.

The other five dependent variables were measured by the following steps: (1) For each dependent variable, the numeric values chosen by each individual respondent for each question, which is five-point Likert scale question, were added and (2) the sum-score for each question was then divided by the total number of questions to obtain an average score. For instance, for acceptance of the HPV vaccination, each individual was asked to answer a total of seven questions; thereafter, the total score of each question was divided by seven. The value of the acceptance score ranged from one to five, and a higher value indicated a higher level of acceptance.

For independent variables, school location was measured as a binary variable (urban vs. suburban). The survey respondent was measured as a nominal variable with three levels, which are mother, father, and others including aunt, grandfather, grandmother, and uncle. Age was used as an ordinal variable with three levels, which are the age under 30, 31–35, and over 35 years. Education was measured as an ordinal variable with four levels, namely primary school or below, secondary school, vocational school, and college or above.

Employment was used as a binary variable (yes vs. no) in which the respondents classified into the group ‘yes’ were employee, owner of business/merchant, government officer, or farmer. Income, which represented the monthly household income in THB, was measured as an ordinal variable. The number of daughters was also used as an ordinal variable. Any childhood vaccines were measured as a binary variable (yes vs. others). Each individual respondent was asked to answer a question ‘my daughter has received other recommended childhood vaccines.’ If respondents answered ‘yes all’ or ‘yes some,’ they were classified into the group ‘yes.’ If respondents answered ‘no’ or ‘not sure,’ they were classified into the group ‘others’.

Religious importance was measured as a binary variable (very/rather important vs. rather little/very little importance). Both alcohol use and smoking were measured as binary variables (regular/irregular vs. non-drinker/smoker). Respondents were classified into the group ‘regular/irregular’ based on if they were weekly, monthly, or non-regular drinkers or smokers. Both health check-up and Pap Smear test were measured as ordinal variables with four levels, which were never, 1- or 2-year interval, 2- or 5-year interval, and more than 5-year interval. Mother’s abnormal Pap Smear and mother’s cervical cancer history were measured as nominal variables with three levels, which were yes, no, and not sure.

Background knowledge of HPV was measured as a binary variable (yes vs. no). Each individual respondent was asked to answer three yes or no questions such as ‘have you ever heard about HPV?’ If the respondents answered ‘no’ to all three questions, they were classified into the group ‘no.’ If not, they were classified into the group ‘yes.’ The variable background knowledge of the HPV vaccine was also addressed in the same manner.

### Statistical analysis

Since all dependent variables were continuous, ordinary least squares (OLS) regression analyses were performed. A total of six OLS analyses were conducted separately. First, we performed an OLS analysis to examine the relationship between the independent variables and knowledge of HPV and cervical cancer (Model 1). Second, four separate OLS analyses were conducted to examine how the knowledge from Model 1 is associated with beliefs about HPV and the HPV vaccine, which includes susceptibility, severity, benefit, and barriers of HPV and the HPV vaccine (Models 2–5). Finally, an OLS analysis was conducted to examine how the knowledge and the beliefs were related to acceptance of the HPV vaccination (Model 6).

Assumptions of the OLS models, particularly multicollinearity and residual normality, were tested. For multicollinearity, both Pearson and Spearman correlation analyses were conducted for continuous variables, and Cramer’s V was calculated for the categorical variables. Modest correlation coefficients obtained from the analyses suggested that multicollinearity did not appear to be problematic. Additionally, tolerance tests in each OLS model did not show multicollinearity to be an issue. For residual normality, we inspected the residual distributions of each OLS model and found that there was no strong violation of the normality assumption. All data management and statistical analyses in this study were performed by using IBM SPSS Statistics version 20 software (S3 File. Data set).

### Ethical considerations

The study was conducted according to the ethical requirements stated in the Declaration of Helsinki [52]. All participants received information before giving their written consent. The participants were informed that participation was voluntary and that they could withdraw participation at any time. They were also informed that only the researchers would have access to the data and that all data would be presented on a group level. The Committee for Research Ethics, Faculty of Social Sciences Humanities, Mahidol University, Nahkon Pathon, Thailand, approved the study. Approval no: 2015/331.2010; MU SSIRB no: 2015/389 (B2).

## Results

In total, 359 (100%) parents completed the questionnaire. Fifty-eight parents were excluded due to incomplete responses. A total of 301 (84%) participants (see Table 1) were included in the final analysis and presented in the results. Almost all of the included parents (99%) were Buddhists.

**Table 1:** Characteristics of the participants with daughters aged 9–12 (n = 301).

Variable		n	(%)
**School location**			
	Urban (Bangkok)	153	50.83
	Suburban (Nakhon Pathom)	148	49.17
**Survey respondent**			
	Mother	240	79.73
	Father	32	10.63
	Others	29	9.63
**Age (years)**			
	Under 30	37	12.29
	31–35	85	28.24
	Over 35	179	59.47
**Education**			
	Primary school or below	111	36.88
	Secondary school	105	34.88
	Vocational school	53	17.61
	College or above	32	10.63
**Employment**			
	Yes	196	65.12
	No	105	34.88
**Income (THB)**^†^			
	Less than 5,000	36	11.96
	5,000–9,999	101	33.55
	10,000–29,999	141	46.84
	More than 30,000	23	7.64
**Number of daughters**			
	Only one	161	53.49
	Two	108	35.88
	Three or more	32	10.63
**Any childhood vaccinations**			
	Yes	230	76.41
	Others	71	23.59
**Importance of Religion (Buddhism)**			
	Very/rather important	273	90.7
	Rather little/very little importance	28	9.3
**Drinking (alcohol)**			
	Yes	72	23.92
	No	229	76.08
**Smoking (tobacco)**			
	Yes	31	10.3
	No	270	89.7
**Health check-up**			
	1- or 2-year interval	131	43.52
	2- or 5-year interval	56	18.6
	More than 5-year interval	28	9.3
	Never	86	28.57
**Mother’s Pap Smear test (n = 240)**			
	1- or 2-year interval	87	28.9
	2- or 5-year interval	60	19.93
	More than 5-year interval	36	11.96
	Never	118	39.2
**Mother’s abnormal Pap Smear (n = 240)**			
	Yes	26	8.64
	No	155	51.5
	Not sure	120	39.87
**Mother’s cervical cancer history (n = 240)**			
	Yes	14	5.98
	No	159	66.45
	Not sure	83	27.57
**Background knowledge of HPV**			
	Yes	157	52.16
	No	144	47.84
**Background knowledge of HPV vaccine**			
	Yes	138	45.85
	No	163	54.15

^†^1 USD = 33.0791THB (September 2017)

As shown in Table 1, the majority (80%) of the participants were the daughter’s mothers, and one out of ten (11%) had college level or above education. For income (income is measured in THB throughout the paper), the majority (80%) of the participants were in the category of 5,000–29,999 THB, while 12% had an income of less than 5,000 THB. Almost one-fourth (24%) were daily smokers, and 10% were regular drinkers. Four out of ten (39%) reported that they had never undergone a Pap smear test. The majority (76%) reported that their daughters had received (all or some) of the recommended childhood vaccinations. For background knowledge about HPV and the HPV vaccine, 52% and 46% reported that they had heard of or been informed about HPV and the HPV vaccine, respectively (Table 1). The information channel included friends/family, media/advertisement, and healthcare professionals.

Table 2 shows the results for parents’ acceptance, beliefs, and knowledge about HPV and the HPV vaccine. As mentioned above in the statistical analysis, the scores ranged from 1–5 points, and a higher score indicated higher acceptance, perceived benefit, barriers, susceptibility, and severity about HPV and the HPV vaccine. The mean of acceptance was 3.52, which indicates that the respondents, on average, had a high acceptance of the HPV vaccine for their daughters. For beliefs, the means of susceptibility and severity were 3.42 and 3.83, while the means of benefits and barriers were 3.41 and 3.14, respectively. For knowledge, the mean was 3.96. This reflects that the respondents, on average, had around 4 correct answers out of the 14 true or false question items regarding HPV and cervical cancer.

**Table 2:** Descriptive statistics of parents’ beliefs and knowledge about HPV and the HPV vaccine (n = 301).

Variables	Mean	Std. Dev.	Median
Acceptance of the HPV vaccination^†^	3.52	0.66	3.57
Susceptibility of HPV and the HPV vaccine^†^	3.42	0.79	3.50
Severity of HPV and the HPV vaccine^†^	3.83	0.77	4.00
Benefit of HPV and the HPV vaccine^†^	3.41	0.60	3.33
Barrier of HPV and the HPV vaccine^†^	3.14	0.45	3.00
Knowledge of HPV and cervical cancer^††^	3.96	3.21	4.00

^†^Scores 1–5

^††^ Scores 0–14

### Results of the OLS regression analysis

Table 3 presents the results of the OLS regression analyses. In model 1 (socio-demographic variables vs. knowledge of HPV and cervical cancer), a significant relationship was found between four variables, including income, importance of religion, background knowledge of HPV, and background knowledge of the HPV vaccine.

**Table 3:** Results from the OLS regression analysis with participants’ characteristics and beliefs and knowledge about HPV and the HPV vaccine in relation to the HBM.

	Knowledge	Susceptibility	Severity	Benefit	Barriers	Acceptability
	(Model 1)	(Model 2)	(Model 3)	(Model 4)	(Model 5)	(Model 6)
**Variables**	Estimate (SE)	Estimate (SE)	Estimate (SE)	Estimate (SE)	Estimate (SE)	Estimate (SE)
Susceptibility						0.13 (0.05)^*^
Severity						0.09 (0.05)
Benefit						0.22 (0.07)^**^
Barriers						0.16 (0.08)
Knowledge of HPV and cervical cancer		0.05 (0.02)^**^	0.06 (0.02)^**^	0.03 (0.01)^**^	0.01 (0.01)	0.03 (0.01)^*^
School Location Urban (vs. Suburban)	0.46 (0.36)	-0.16 (0.09)	<0.01 (0.09)	-0.17 (0.07)^*^	-0.05 (0.06)	0.08 (0.07)
Survey Respondent (Mother^a^)						
Father	-0.04 (0.65)	0.22 (0.17)	<0.01 (0.17)	0.21 (0.12)	0.08 (0.10)	0.14 (0.13)
Others	0.31 (0.64)	0.14 (0.16)	-0.18 (0.17)	0.05 (0.12)	0.05 (0.10)	0.24 (0.13)
Age (Under 30^a^)						
31–35	-0.07 (0.59)	-0.14 (0.15)	-0.39 (0.15)^*^	-0.12 (0.11)	-0.08 (0.09)	0.15 (0.12)
Over 35	-0.20 (0.56)	-0.14 (0.14)	-0.47 (0.14)^**^	-0.10 (0.11)	-0.16 (0.09)	0.17 (0.11)
Education (Primary school or below^a^)						
Secondary school	-0.18 (1.31)	-0.21 (0.34)	-0.27 (0.34)	-0.18 (0.25)	-0.03 (0.20)	-0.18 (0.26)
Vocational school	0.31 (1.34)	-0.17 (0.34)	-0.37 (0.35)	-0.18 (0.26)	0.05 (0.21)	-0.12 (0.27)
College or above	-0.17 (1.37)	-0.24 (0.35)	-0.26 (0.35)	-0.30 (0.26)	0.08 (0.21)	-0.13 (0.27)
Employment Yes (vs. No)	0.54 (1.28)	0.09 (0.33)	0.16 (0.33)	0.00 (0.24)	-0.16 (0.20)	0.18 (0.25)
Income THB (Less than 5,000^a^)						
5,001–9,999	0.60 (0.59)	0.18 (0.15)	-0.09 (0.15)	-0.26 (0.11)^*^	-0.14 (0.09)	0.05 (0.12)
10,000–29,999	0.20 (0.57)	0.16 (0.15)	0.04 (0.15)	-0.21 (0.11)	-0.13 (0.09)	0.07 (0.12)
More than 30,000	1.86 (0.83)^*^	0.05 (0.21)	<0.01 (0.22)	0.07 (0.16)	-0.12 (0.13)	-0.10 (0.17)
Number of daughter (Only one^a^)						
Two	0.15 (0.38)	0.06 (0.10)	0.01 (0.10)	0.05 (0.07)	0.10 (0.06)	-0.03 (0.08)
Three or more	0.11 (0.58)	0.38 (0.15)^*^	0.10 (0.15)	0.18 (0.11)	0.31 (0.09)^**^	-0.07 (0.12)
Any childhood vaccinations Yes (vs. Others)	0.73 (0.42)	-0.04 (0.11)	-0.02 (0.11)	0.04 (0.08)	0.18 (0.07)^**^	0.09 (0.09)
Importance of Religion Very/rather important (vs. Rather little/very little importance)	1.67 (0.61)^**^	0.43 (0.16)^**^	0.08 (0.16)	0.37 (0.12)^**^	0.05 (0.10)	0.12 (0.13)
Drinking Regular/irregular (vs. Non-drinker)	-0.30 (0.43)	-0.23 (0.11)^*^	-0.14 (0.11)	0.02 (0.08)	-0.01 (0.07)	0.02 (0.09)
Smoking Regular/irregular (vs. Non-smoker)	0.53 (0.62)	0.02 (0.16)	-0.21 (0.16)	-0.06 (0.12)	0.08 (0.10)	-0.28 (0.12)^*^
Health check-up (Never^a^)						
1- or 2-year interval	0.10 (0.45)	0.09 (0.12)	0.02 (0.12)	-0.07 (0.09)	-0.03 (0.07)	-0.25 (0.09)^**^
2- or 5-year interval	0.28 (0.57)	0.25 (0.15)	0.37 (0.15)^*^	0.09 (0.11)	0.07 (0.09)	-0.16 (0.11)
More than 5-year interval	0.07 (0.69)	0.10 (0.18)	<0.01 (0.18)	-0.02 (0.13)	<0.01 (0.11)	-0.48 (0.14)^**^
Mother's Pap Smear test (Never^a^)						
1- or 2-year interval	0.93 (0.62)	-0.04 (0.16)	-0.01 (0.16)	0.13 (0.12)	0.06 (0.10)	-0.01 (0.12)
2- or 5-year interval	0.26 (0.66)	-0.05 (0.17)	0.03 (0.17)	0.11 (0.13)	0.05 (0.10)	0.01 (0.13)
More than 5-year interval	-0.30 (0.70)	-0.29 (0.18)	0.10 (0.18)	-0.06 (0.13)	0.08 (0.11)	0.13 (0.14)
Mother's abnormal Pap Smear (Yes^a^)						
No	0.21 (0.67)	-0.17 (0.17)	-0.02 (0.17)	0.04 (0.13)	-0.01 (0.10)	-0.10 (0.13)
Not sure	-0.02 (0.82)	0.08 (0.21)	0.01 (0.21)	0.12 (0.16)	0.17 (0.13)	-0.03 (0.16)
Mother's cervical cancer history (Yes^a^)						
No	-0.81 (0.75)	0.18 (0.19)	0.14 (0.19)	-0.04 (0.14)	<0.01 (0.12)	-0.26 (0.15)
Not sure	-0.74 (0.80)	-0.17 (0.21)	0.08 (0.21)	-0.15 (0.15)	-0.09 (0.12)	-0.24 (0.16)
Background Knowledge of HPV Yes (vs. No)	1.12 (0.42)^**^	0.01 (0.11)	-0.08 (0.11)	0.22 (0.08)^**^	0.04 (0.07)	-0.08 (0.09)
Background Knowledge of HPV vaccines Yes (vs. No)	1.88 (0.40)^**^	0.18 (0.11)	<0.01 (0.11)	0.01 (0.08)	-0.07 (0.06)	0.17 (0.08)^*^

^a^ = Reference value

* = Significant at 0.05

** = Significant at 0.01

SE = Standard Error

For income, participants with more than 30,000 THB were positively related to knowledge, compared to those with less than 5,000 THB. This indicated that knowledge of HPV and cervical cancer was significantly higher in participants with a high-income level than those with a low-income level. However, participants with a middle-income level (i.e., participants with 5,001–9,999 THB and with 10,000–29,999 THB) were not significantly related to knowledge compared to those with a low-income level (i.e., participants with less than 5,000 THB).

Importance of religion was positively associated with knowledge, with the coefficient equal to 1.67. Participants who reported that religion was ‘very/rather’ important had greater knowledge of HPV and cervical cancer than those who reported that religion was of ‘rather little/very little’ importance. Lastly, both background knowledge of HPV and the HPV vaccine were positively related to knowledge of HPV and cervical cancer, with the coefficients equal to 1.12 and 1.88, respectively. This indicated that knowledge about HPV and cervical cancer was significantly higher in participants who had heard of or been informed about HPV or the HPV vaccine than those who had not.

In models 2–5 (knowledge of HPV and cervical cancer vs. belief of HPV and the HPV vaccine), knowledge was found to be positively associated with susceptibility, severity, and benefit, with the coefficient equal to 0.05, 0.06, and 0.03, respectively. It meant that participants with greater knowledge of HPV and cervical cancer had higher susceptibility, severity, or benefit of HPV and the HPV vaccine. However, the relationship between knowledge and barriers was positive but not statistically significant.

For socio-demographic variables, school location was negatively related to benefit. Specifically, participants who had daughters attending the schools in Bangkok had lower benefit than the school in the suburb. Age was negatively associated with severity. Older participants, those who were over 31, had lower severity than younger participants, those who were under 30. For income, participants with 5,001–9,999 THB had lower benefit than those with less than 5,000 THB.

Importance of religion was positively related to both susceptibility and benefit. Specifically, participants who reported that religion was ‘very/rather’ important had higher susceptibility and benefit than those who reported that religion was of ‘rather little/very little’ importance. For alcohol use, participants who were ‘regular/irregular’ drinkers reported lower susceptibility than those who were non-drinkers. Lastly, for background knowledge of HPV, participants who had heard of or been informed about HPV had higher benefit than those who had not.

Finally, model 6 presents the results of the OLS analysis to investigate how knowledge of HPV and cervical cancer and belief of HPV and the HPV vaccine are associated with acceptance of the HPV vaccination. Knowledge was positively related to acceptance, with the coefficient equal to 0.03. Participants with greater knowledge had higher acceptance of the HPV vaccination. For belief, participants with higher susceptibility were found to have higher acceptance of the HPV vaccination.

Additionally, for socio-demographic variables, participants who were ‘regular/irregular’ smokers had lower acceptance of the HPV vaccination than those who were non-smokers. For background knowledge of the HPV vaccine, participants who had heard of or been informed about the HPV vaccine had higher acceptance than those who had not.

## Discussion

Parents are the ones that decide whether or not to vaccinate their children against HPV; the decision is complex, and several factors are important. On an individual level, the decision is based on attitudes, beliefs, knowledge, subjective norms, socio-demographics, as well as cultural and religious aspects. While on a national level, governmental policies and access to adequate health services (such as health check-ups, vaccination- and screening programs) are significant factors. There are gender differences in the decision-making process and in the level of knowledge one has about HPV and the HPV vaccination. Previous research findings on whether religion has an influence on beliefs and acceptance of the HPV vaccination are inconsistent. HBM is a common theoretical framework used in studies about the behavioral aspects of HPV and the HPV vaccination from a public health perspective. Thus, this study aimed to examine parents’ knowledge, beliefs, and acceptance of HPV and the HPV vaccine for their young daughter, in relation to their knowledge, socio-demographics, health behavior, and religious beliefs.

In summary, our results showed that parents with greater knowledge of HPV perceived: a higher susceptibility for contracting HPV, HPV as being more severe, and more benefits about the vaccines protective effect against cervical cancer. Moreover, parents with greater knowledge had higher acceptance of the vaccine. There were also differences in knowledge due to the socio-demographic variables; parents in the higher-income group had greater knowledge of HPV and the HPV vaccine compared to parents in the lower-income bracket. Finally, parents who reported religion as being more important perceived higher susceptibility for an HPV-infection or cancer and perceived more benefits with the vaccination against HPV. In contrast to our hypotheses, parents who had a lower-income perceived more benefits compared to parents with a higher income. Almost 40% of the participating mothers had never undergone a cervical cancer screening test (Pap smear).

Interestingly, participants who reported religion as being important had greater knowledge of HPV and cervical cancer than those who reported that religion was less important. This is an important finding since religion has a vital impact on the Thai society, and over 99% of the included parents were Buddhists. More than half of the participants (52%) had heard of HPV, and 45% reported that they had been informed about the HPV vaccine. The parents had received the information from friends and family, media/advertisement, and healthcare professionals. Information about the HPV vaccine might be a trigger, cues to action, for the individual health behavior; thus, healthcare professionals’ recommendations are important for making a decision about the HPV vaccine [10, 53, 54].

The overall knowledge about HPV and the vaccine was very low. The low knowledge is not surprising since the vaccine is not yet implemented in the national vaccination program, and the population does not receive information about the HPV vaccine on a regular basis. Low knowledge about HPV and the vaccine is common among parents worldwide [11, 20–25, 39] as well as among the population in Thailand [55]. It has been emphasized that parents need to be provided with adequate information in order to make an informed decision about whether or not to vaccinate their child against HPV [28, 56]. According to the HBM, knowledge is a moderating factor for individual beliefs and the actual health behavior [46].

We found that religion was positively related to both susceptibility and benefit (i.e., respondents who reported that religion was important had higher susceptibility and benefit than those who reported that religion was less important). It is probable that those parents who reported religion as being of importance have a strong belief in “Karma” i.e., the misfortune, good or bad things that happened in life are the result from the previous life, which they cannot control so that the risk of cancer can be real and the vaccine will help to resolve it [42, 43]. The importance of religion in relation to the beliefs about the HPV vaccine varies, according to previous studies [10, 20, 34, 35, 38, 39]. Studies undertaken in Canada and Sweden indicate that religion does not influence one’s beliefs about the vaccine [10, 20]. Comparable results are also found among Indonesian parents [39]. In contrast, a U.S. study found that parents who attended religious services had less favorable beliefs and lower vaccine acceptance compared to parents with no religious affiliation [34]. Similar results have been found among parents in a religious community in the U.K. [35].

Participants who had heard of or been informed about HPV (background knowledge) perceived a higher benefit of the vaccine than those who had not. Consequently, providing information about HPV and the HPV vaccine is essential for knowledge and awareness of the virus and the vaccine and can contribute to more favorable beliefs. There were also differences in the beliefs, depending on the socio-demographic variables. Parents with a daughter in the schools in Bangkok, (urban area) had relatively low benefit of the vaccine compared to those who had daughters attending the school in the suburban area. This is in line with research from Botswana and India, which showed that parents outside of cities were more in favor of vaccinating their daughters against HPV than those in the urban areas. Parents’ favorable beliefs about the HPV vaccine is a key factor for acceptance of the vaccine, as discussed in previous research in different contexts [10, 20, 26, 27, 56].

Background knowledge of the HPV vaccine was positively related to acceptance, indicating the importance of parents having knowledge about the vaccine before implementation. The acceptance of the HPV vaccine was high among the included parents. The majority had accepted previous childhood vaccinations, which is important since vaccine hesitancy is a growing challenge globally [57]. Research shows that acceptance of previous childhood vaccinations is associated with parental acceptance of the HPV vaccination [10, 20, 27, 39]. Thailand has high coverage of childhood vaccinations, and countries with well-functioning national vaccination programs, especially countries with school-based vaccinations, have a higher coverage of the HPV vaccination [9].

The Global Vaccine Action Plan is a roadmap to prevent millions of deaths through more equitable access to vaccines. One key element is ensuring that everyone can have access to vaccines and afford to pay for them. Thailand has a well-functioning vaccination program that is free of charge, reaching high coverage of over 95%. To implement vaccination against HPV in the national vaccination program for children would be in line with the Thai Ministry of Public Health’s policies for the population to be protected from vaccine-preventable diseases. It would be beneficial for the individual as well as for the public health and would decrease the high incidence of cervical cancer, thus, saving lives.

### Strengths and limitations

The study was conducted in Thailand, a country with the highest incidence of cervical cancer worldwide. The study is undertaken in different socio-demographic and geographical areas, both city and rural areas, and includes a diverse range of parents of different beliefs and socio-economic status. Another strength was the theoretical framework of the HBM, which is a systematic way to explain a person’s health beliefs [46]. The design was considered appropriate since there is no previous knowledge about Thai parents’ beliefs and acceptance of the HPV vaccination. However, the cross-sectional design cannot provide information about causality regarding parental acceptance of the HPV vaccination.

The majority of the participants were mothers. One reason for this might be that in Thailand mothers are usually the decision-makers about the child’s health. Research also shows that mothers are the decision-makers regarding the HPV vaccination [15, 16]. The parents completed the self-reported questionnaire in their homes, and self-reported data must always be interpleaded with caution. Furthermore, we purposively selected three schools, and such purposive sampling method with a small sample size may include overgeneralization. Thus, this issue should be taken into consideration in a future study. In addition, we recommend longitudinal analysis in order to decrease the issue of simultaneity that our OLS analyses include.

### Conclusions

We found associations between parents’ knowledge, beliefs, and acceptance of the HPV vaccination for their young daughter, in relation to their socio-demographic variables and religious beliefs. Parents who reported religion as being important were more in favor of the HPV vaccination compared to parents who reported religion as being of less importance. Four out of ten of the included mothers had never undergone a cervical cancer screening but had accepted previous childhood vaccinations for their daughters. Acceptance of the vaccine was high, and we believe our results are promising for future implementation of the HPV vaccination in the national childhood vaccination program in Thailand.

## Supporting information

S1 File STROBE checklist. (DOCX)

S2 File Questionnaire. (DOCX)

S3 File Data set. (SAV)
