# Correction: Parents' knowledge, beliefs, and acceptance of the HPV vaccination in relation to their socio-demographics and religious beliefs: A cross-sectional study in Thailand

**DOI:** 10.1371/journal.pone.0196437

**Published:** 2018-04-19

**Authors:** Maria Grandahl, Seung Chun Paek, Siriwan Grisurapong, Penchan Sherer, Tanja Tydén, Pranee Lundberg

The second author’s name appears incorrectly in the citation. The correct citation is: Grandahl M, Paek SC, Grisurapong S, Sherer P, Tydén T, Lundberg P (2018) Parents’ knowledge, beliefs, and acceptance of the HPV vaccination in relation to their socio-demographics and religious beliefs: A cross-sectional study in Thailand. PLoS ONE 13(2): e0193054. https://doi.org/10.1371/journal.pone.0193054
