# Supplementary material for: Parents’ knowledge, beliefs, and acceptance of the HPV vaccination in relation to their socio-demographics and religious beliefs: A cross-sectional study in Thailand
Source: PLoS One. 2018 Feb 15;13(2):e0193054. doi: 10.1371/journal.pone.0193054 (PMC5814087; doi:10.1371/journal.pone.0193054)
Supplement: S2 File — (DOCX) [file pone.0193054.s002.docx]

**Knowledge, belief and acceptance among parents in Thailand related to Human Papilloma Virus Vaccination of their daughters**

This questionnaire is aimed at investigating maternal knowledge, belief and acceptance among parents in Thailand related to Human Papilloma Virus Vaccination of their daughters. Please mark by “X” your answer of each questions of the questionnaire.

1. You are the girl’s

1□ Mother 2 □ Father 3 □ Someone else, 3.1 please specify

2. Your Age

1□ Less than 26 2□ 26-30 3□ 31-35 4□ More than 35

3. Your religion

1□ Buddhist 2□ Christian 3□ Muslim

4□ Other, 4.1 please specify …………………………………………

4. Level of education

1□ Primary school 2□ Secondary school

3□ Vocational school 4□ University/college

5□ Others, 5.1 please specify ……………………………..

5. Current occupation

1□ Unemployed 2□ Governmental officer

3□ Owner of a business or merchant 4□ Employee

5□ Labour 6□ Farmer

7□ Others, please specify ……………………………..

6. Monthly household income (bath)

1□ Less than 5,000 2□ 5,000-9,999

3□ 10,000-29,000 4□ 30,000-49,999

5□ More than 50,000

7. Number of daughters

□ 1 □ 2 □ 3 or more

8. Age/ages of daughter/daughters

Ages of daughter No 1 ( who is studying in this school)……..

Ages of daughter No 2…….

Ages of daughter No 3…….

9. My daughter has received other recommended childhood vaccines

1□ Yes all 2□ Yes some 3 □No 4□ Unsure

10. How important is your religion to you?

1□ Very important 2□ Rather important

3□ Neither important nor unimportant 4□ Rather little important

5□ Very little important

11. Lifestyle related to health concerning

11.1 Alcohol drinking: 1□ Weekly 2□ Monthly

3□ Not regular 4□ Never

11.2 Smoking: 1□ 10-20/day 2□ Less than 10/day

3□ Not regular 4□ Never

11.3 Health check: 1□ Never 2□ More than 1-2 years interval

3□ 2-5 years interval 4□ More than 5 years interval

11.4Pap smear (female): 1□ Never 2□ more than 1-2 years interval

3□ 2-5 years interval 4□ more than 5 years interval

12. If you are the mother of the girl, please answer this question.

I have had an abnormal Pap smear myself 1□ Yes 2□ No 3□ Don’t know

13. If you are the mother of the girl, please answer this question.

History of cervical cancer (myself or relative) 1□ Yes 2□ No 3□ Don’t know

14. Background knowledge about HPV and HPV vaccine

|  |  | 1Yes | 2 No |
| --- | --- | --- | --- |
| a) | Have you ever heard about HPV? | □ | □ |
| b) | Have you ever asked for information about HPV? | □ | □ |
| c) | Have you ever been informed about HPV by physician/nurse? | □ | □ |
| d) | From where have you heard about HPV? |  |  |
|  | - Friends | □ | □ |
|  | - Family/relatives | □ | □ |
|  | - Advertisement | □ | □ |
|  | - Internet | □ | □ |
|  | - Mass media (TV, radio, newspaper) | □ | □ |
| e) | Have you ever heard about HPV vaccine? | □ | □ |
| f) | Have you ever asked for information about HPV vaccine? | □ | □ |
| g) | Have you ever been informed about HPV vaccine by physician/nurse? | □ | □ |
| h) | From where have you ever heard about HPV vaccine? |  |  |
|  | - Friends | □ | □ |
|  | - Family/relatives | □ | □ |
|  | - Advertisement | □ | □ |
|  | - Internet | □ | □ |
|  | - Mass media (TV, radio, newspapaer) | □ | □ |

15. Knowledge about HPV and cervical cancer

|  |  | 1True | 2False | 3Don’t know |
| --- | --- | --- | --- | --- |
| a) | HPV infection is contracted by sexual contact. | □ | □ | □ |
| b) | People can transmit HPV to their partner(s) even if they have no symptoms of HPV infection. | □ | □ | □ |
| c) | Having multiple sexual partners’ increases risk of HPV infection. | □ | □ | □ |
| d) | Sex at early age increases risk of HPV infection. | □ | □ | □ |
| e) | Genital warts are caused by HPV infection. | □ | □ | □ |
| f) | Most people with genital HPV have no visible signs or symptoms. | □ | □ | □ |
| g) | HPV infection can be prevented by vaginal douching after intercourse. | □ | □ | □ |
| h) | HPV infection can be treated by antibiotics. | □ | □ | □ |
| i) | Smoking increases risk of cervical cancer. |  |  |  |
| j) | HPV infection can cause cervical cancer. | □ | □ | □ |
| k) | Cervical cancer symptoms commonly present with vaginal discharge or bleeding even in the early stages of disease. | □ | □ | □ |
| l) | Cervical cancer can possibly cause bleeding after sex. | □ | □ | □ |
| m) | A Pap smear is only indicated in women with vaginal discharge or bleeding. | □ | □ | □ |
| n) | Unmarried women are not supposed to have a Pap smear. | □ | □ | □ |

16. Beliefs about HPV and HPV vaccine

|  | |  | 1Strongly disagree | 2Disagree | 3Unsure | 4Agree | 5Strongly agree |
| --- | --- | --- | --- | --- | --- | --- | --- |
| a) | There is a risk for young women to contract HPV | | □ | □ | □ | □ | □ |
| b) | There is a risk for young women to contract cervical cancer | | □ | □ | □ | □ | □ |
| c) | HPV infection is a serious health concern | | □ | □ | □ | □ | □ |
| d) | Cervical cancer is a serious disease | | □ | □ | □ | □ | □ |
| e) | The HPV vaccine is effective in preventing condyloma | | □ | □ | □ | □ | □ |
| f) | The HPV vaccine is effective in preventing cervical cancer | | □ | □ | □ | □ | □ |
| g) | I have trust in the HPV vaccination | | □ | □ | □ | □ | □ |
| h) | The HPV vaccine can cause adverse effects | | □ | □ | □ | □ | □ |
| i) | It is problematic that HPV vaccination requires three injections | | □ | □ | □ | □ | □ |
| j) | The efficiency of HPV vaccine is unclear | | □ | □ | □ | □ | □ |
| k) | The HPV vaccine is harmful | | □ | □ | □ | □ | □ |
| l) | Women who have been HPV vaccinated should have Pap smear annually | | □ | □ | □ | □ | □ |
| m) | HPV vaccination decreases c condom use | | □ | □ | □ | □ | □ |
| n) | HPV vaccination causes my daughter to be sexually active early | | □ | □ | □ | □ | □ |
| o) | HPV vaccination increases number of sexual partners | | □ | □ | □ | □ | □ |
| p) | HPV vaccination increases awareness of sexually transmitted diseases | | □ | □ | □ | □ | □ |

17. Acceptability of HPV vaccination

|  |  | 1Strongly  disagree | 2Disagree | 3Unsure | 4Agree | 5Strongly agree |
| --- | --- | --- | --- | --- | --- | --- |
| a) | Do you agree if Ministry of Public Health is going to offer HPV vaccine for free to 9-11 years old girls? | □ | □ | □ | □ | □ |
| b) | Do you agree with the policy of giving vaccination to every child? | □ | □ | □ | □ | □ |
| c) | Do you consider vaccinating yourself (for mother)? | □ | □ | □ | □ | □ |
| d) | Will you accept active HPV vaccination of your daughter? | □ | □ | □ | □ | □ |
| e) | If your daughter is not in the target group do you still want to pay for vaccinating her? | □ | □ | □ | □ | □ |
| f) | If the government offers HPV vaccination for free, I will vaccinate my daughter | □ | □ | □ | □ | □ |
| g) | Now HPV vaccine costs 6000-6900 baht per course, yet I will vaccinate my daughter | □ | □ | □ | □ | □ |
| h) | I don’t have enough information about HPV vaccine to decide whether to give it to my daughter | □ | □ | □ | □ | □ |
| i) | HPV vaccine is so new that I want to wait a while before deciding if my daughter should get it | □ | □ | □ | □ | □ |

18. If you have any comments please write it down to us.

…………………………………………………………………………………………………...

…………………………………………………………………………………………………...

Thank you for your answer!
